# Factors Influencing Clinicians’ Willingness to Prescribe Pre-exposure Prophylaxis for Persons at High Risk of HIV in China: Cross-sectional Online Survey Study

**DOI:** 10.2196/24235

**Published:** 2021-06-04

**Authors:** Sitong Cui, Haibo Ding, Xiaojie Huang, Hui Wang, Weiming Tang, Sequoia I Leuba, Zehao Ye, Yongjun Jiang, Wenqing Geng, Junjie Xu, Hong Shang

**Affiliations:** 1 NHC Key Laboratory of AIDS Immunology (China Medical University) National Clinical Research Center for Laboratory Medicine The First Affiliated Hospital of China Medical University Shenyang, Liaoning Province China; 2 Key Laboratory of AIDS Immunology Chinese Academy of Medical Sciences Shenyang China; 3 Key Laboratory of AIDS Immunology of Liaoning Province Shenyang China; 4 Collaborative Innovation Center for Diagnosis and Treatment of Infectious Diseases Hangzhou China; 5 Center for Infectious Diseases Beijing You'an Hospital Capital Medical University Beijing China; 6 Shenzhen Third People’s Hospital Shenzhen China; 7 Project-China, University of North Carolina at Chapel Hill Guangzhou China; 8 Department of Epidemiology University of North Carolina at Chapel Hill Chapel Hill, NC United States

**Keywords:** WeChat, pre-exposure prophylaxis, clinicians, willingness to prescribe, HIV prevention, China

## Abstract

**Background:**

Pre-exposure prophylaxis (PrEP) is an effective HIV prevention measure. Clinicians play a crucial role in PrEP implementation, and their knowledge, attitudes, and career experience may affect their willingness to prescribe PrEP. However, little is known about the attitudes and willingness of clinicians to prescribe PrEP in countries without PrEP-specific guidelines.

**Objective:**

We aimed to determine the factors associated with clinicians being willing to prescribe PrEP in China.

**Methods:**

Between May and June 2019, we conducted an online cross-sectional survey of clinicians in 31 provinces across the six administrative regions in China on the WeChat smartphone app platform. Multivariable logistic regression was used to determine factors associated with willingness to prescribe PrEP.

**Results:**

Overall, 777 HIV clinicians completed the survey. Most of the respondents had heard of PrEP (563/777, 72.5%), 31.9% (248/777) thought that PrEP was extremely effective for reducing the risk of HIV infection, and 47.2% (367/777) thought that it was necessary to provide PrEP to high-risk groups. After adjusting for age, gender, ethnicity, and educational background of the clinicians, the following factors signiﬁcantly increased the odds of the clinicians being willing to prescribe PrEP: having worked for more than 10 years, compared to 5 years or less (adjusted odds ratio [aOR] 2.82, 95% CI 1.96-4.05); having treated more than 100 patients living with HIV per month, compared to 50 patients or fewer (aOR 4.16, 95% CI 2.85-6.08); and having heard of PrEP (aOR 7.32, 95% CI 4.88-10.97). Clinicians were less likely to be willing to prescribe PrEP if they were concerned about poor adherence to PrEP (aOR 0.66, 95% CI 0.50-0.88), the lack of PrEP clinical guidelines (aOR 0.47, 95% CI 0.32-0.70), and the lack of drug indications for PrEP (aOR 0.49, 95% CI 0.32-0.76).

**Conclusions:**

About half of all clinicians surveyed were willing to prescribe PrEP, but most surveyed had a low understanding of PrEP. Lack of PrEP clinical guidelines, lack of drug indications, and less than 11 years of work experience were the main barriers to the surveyed clinicians’ willingness to prescribe PrEP. Development of PrEP clinical guidelines and drug indications, as well as increasing the availability of PrEP training, could help improve understanding of PrEP among clinicians and, thus, increase the number willing to prescribe PrEP.

## Introduction

Pre-exposure prophylaxis (PrEP) is a highly effective preventative strategy for HIV; through a combination of HIV approaches recommended by the World Health Organization (WHO), PrEP can reduce HIV transmission [1]. PrEP strategies involve key HIV-negative populations who take antiretroviral medications and attend routine visits with an HIV clinician in order to prevent HIV transmission. Recent clinical trials among men who have sex with men (MSM) have shown that daily oral PrEP and on-demand PrEP can prevent 86% and 96% of HIV infections, respectively [2]. Tenofovir disoproxil fumarate/emtricitabine (TDF/FTC; brand name Truvada) has been approved by the WHO for PrEP use among adults and adolescents at risk of HIV [3,4]. About 40 countries around the world have incorporated PrEP into their health systems, 10 of which have carried out nationwide programs [2]. The other 30, including China, are implementing pilot projects exploring the acceptability, effectiveness, and cost of PrEP [2,5]. At present, only a few countries have provided support on implementing this new strategy through country-specific guidelines for clinicians [3,6].

Before prescribing PrEP, physicians must assess the HIV risk of the patient, their likely adherence, and any potential side effects in order to ensure effective use of PrEP [7-10]. PrEP is most effective at reducing HIV risk when targeted toward high-risk populations [6]. Assessing likely adherence is crucial, as patients with low adherence to PrEP did not have a reduction in HIV risk [11]. In addition, as some high-risk populations have anxiety and depression [12], any potential side effects of PrEP must be assessed by the clinician prior to prescribing PrEP. Thus, any support in making these judgments before prescribing is needed.

One potential method to support clinicians in prescribing PrEP is through providing clinical guidelines to standardize HIV risk assessment. In countries where national PrEP clinical guidelines have been issued, physicians weigh PrEP-related knowledge, attitudes, and experiences when making their decision to prescribe PrEP [13,14]. Some developed countries (eg, the United States, the United Kingdom, and Canada) have examined the attitudes and intentions to prescribe PrEP among clinicians through investigations [15-22]. However, in low- and middle-income countries that do not have national PrEP clinical guidelines, clinicians’ knowledge and willingness to prescribe PrEP has not previously been well-reported.

Despite the high burden caused by the HIV epidemic, China has been slower to implement PrEP compared to other developed countries [2], and few studies have evaluated PrEP use in China. The National Health Commission in China has stated that the country needs to promote PrEP implementation by carrying out nationwide pilot work. The Chinese Center for Disease Control and Prevention has established pilot projects in seven provinces, running from October 2008 to November 2019, to study the feasibility of using PrEP in key populations [23]. In addition, China Medical University carried out real-world research of PrEP use among MSM in Shenyang, Beijing, Chongqing, and Shenzhen starting in December 2018; this research concluded in December 2020 [24]. In addition to these real-world studies on PrEP implementation, several studies have also shown that Chinese MSM are very willing to use PrEP [25,26]. However, the current proportion of PrEP prescriptions in key populations in China is less than 1%, much lower than that in other developed countries (9.5% in the United States and 2.5% in Australia) [27]. Clinicians may be reluctant to prescribe PrEP because of limited understanding, thus leading to this low percentage of prescriptions among key groups in China.

To evaluate PrEP-related attitudes among Chinese clinicians, we conducted a nationwide online survey investigating PrEP-related awareness and attitudes, experience with PrEP, and potential perceived barriers to being willing to prescribe PrEP.

## Methods

### Respondents and Procedures

Between May and June 2019, we conducted a nationwide anonymous online survey among Chinese HIV clinicians in 31 provinces across six administrative regions. The questionnaire was designed based on current literature of HIV prevention using PrEP in China and with the support of subject matter experts. Clinicians with clinical medical qualifications were eligible for the study if they were currently working in a position at an infectious disease or general hospital that included treating patients living with HIV. We reached out to HIV clinicians through the two largest professional work groups in China on WeChat, the most popular social media app in China: the *National clinicians group focusing on HIV/AIDS* and the *National physician platform for the communication of difficult HIV/AIDS cases*. The administrators of these two WeChat groups confirmed the clinical identities and specialties of their members, and removed any members with nonclinical identifications. Thus, 937 HIV clinicians were eligible for this survey (Multimedia Appendix 1). After conducting a pilot study with 50 eligible participants by convenience sampling, which was included in the final results, we evaluated the accuracy and reliability of the questionnaire and modified the survey accordingly. To recruit participants from the remaining eligible clinicians, we posted a brief description of the survey, including the purpose and significance, and links to the questionnaire. The inclusion criteria for this survey were as follows: (1) 18 years of age or older, (2) practicing in an HIV-related medical institution, and (3) treated at least one person living with HIV over the past year. Based on the open ID of WeChat, each individual was allowed to access the online survey only once and the answers could be reviewed or changed before submission. We confirmed the identity of participants through collected self-reported information about medical background and relevant experience. After submission of the completed questionnaire, we provided a subsidy of 30 Yuan (US $4.50) to participants to compensate for the time they spent completing the survey (ie, about 6 to 10 minutes). We used self-reported contact information only to deliver the subsidy. Ethical approval was obtained from the Institutional Review Board of the First Affiliated Hospital of China Medical University ([2019]2015-138-9). In our analysis, we followed the CHERRIES (Checklist for Reporting Results of Internet E-Surveys) guidelines (Multimedia Appendix 2).

### Measures

After providing online informed consent, clinicians completed a voluntary anonymous survey. The questionnaire asked about sociodemographic characteristics; medical background; PrEP-related knowledge, attitudes, and experience; and barriers to prescribing PrEP (Multimedia Appendix 3). 

The primary outcome of this study was the percentage of clinicians willing to prescribe PrEP to key populations based on their response—*yes* or *no*—to the following question: “Do you think it is necessary for clinicians to provide PrEP to HIV high-risk populations to reduce HIV infections?” The questionnaire also asked about the location, administrative level, and type of hospital where they practiced; their academic title; length of their career; and the average number of patients living with HIV treated in the past month. We then assessed PrEP-related knowledge: whether participants had ever heard of PrEP, their understanding of PrEP, how effective they thought PrEP was for reducing the risk of HIV infections, and whether clinical guidelines for PrEP were available in China. We also asked how often they recommend PrEP to MSM, heterosexual males, heterosexual females, and serodiscordant couples. Respondents were also asked how often they saw high-risk groups actively seeking a PrEP prescription in the past 6 months and potential barriers to them prescribing PrEP.

### Statistical Analysis

Continuous variables were categorized for analysis. Age was grouped into five categories (25, 26-39, 40-49, 50-59, and 60 years), length of career was grouped into three categories (5, 6-10, and 11 years), and average number of patients living with HIV treated per month was also grouped into three categories (50, 51-100, and 101 patients). We described the distribution of the variables by presenting the frequency and percentage. We then conducted univariable and multivariable logistic regression to determine predictors of clinicians being willing to prescribe PrEP and presented crude odds ratios (ORs) and adjusted ORs (aORs) with the corresponding 95% CIs. If there were no clinicians in a specific cell in a comparison, 0.5 was added to all cells to compute the OR [28]. Significant predictors in the univariable analysis were included in the multivariable logistic regression. We adjusted for age, gender, ethnicity, and educational background of the clinician in the multivariable model. All statistical analyses were performed with SPSS, version 25.0 (IBM Corp). A two-tailed *P* value of less than .05 was considered statistically significant.

## Results

### Demographic and Medical Background Characteristics

A total of 777 eligible clinicians completed the survey, which represented a response rate of 82.9% (777/937) (Multimedia Appendix 1). Among these 777 survey responses, 50 (6.4%) were from the pilot survey. The median age of the participants was 42 years (IQR 36-48), around half were female (417/777, 53.7%), the most common ethnicity was Han (712/777, 91.6%), and most had a bachelor’s degree or above (743/777, 95.6%). The highest proportion of participants were from South Central China (201/777, 25.9%). The distribution of HIV clinicians surveyed at the province level and economic level is included in Multimedia Appendix 4. Approximately half of the participants worked in infectious disease hospitals (394/777, 50.7%), and 54.2% (421/777) were deputy chief physicians or chief physicians. Slightly more than half (432/777, 55.6%) had worked for more than 5 years, and 59.3% (461/777) treated an average of 50 or fewer patients living with HIV per month (Table 1).

**Table 1:** Demographic and medical background characteristics of clinicians.

Characteristic			Value (N=777), n (%)		
**Demographic characteristic**					
	**Age (years)**				
		≤25	3 (0.4)		
		26-39	302 (38.9)		
		40-49	331 (42.6)		
		50-59	131 (16.9)		
		≥60	10 (1.3)		
	**Ethnicity**				
		Han	712 (91.6)		
		Non-Han	65 (8.4)		
	**Gender**				
		Male	360 (46.3)		
		Female	417 (53.7)		
	**Administrative region of China^a^**				
		South Central	201 (25.9)		
		Southwest	199 (25.6)		
		Northeast	164 (21.1)		
		East	116 (14.9)		
		North	71 (9.1)		
		Northwest	26 (3.3)		
	**Education level**				
		Technical secondary school	5 (0.6)		
		Junior college	29 (3.7)		
		Bachelor’s degree or above	743 (95.6)		
**Medical background and experience**					
	**Type of hospital**				
		General	383 (49.3)		
		Infectious disease	394 (50.7)		
	**Academic title**				
		General physician	82 (10.6)		
		Attending doctor	274 (35.3)		
		Deputy chief physician	221 (28.4)		
		Chief physician	200 (25.7)		
	**Specialization**				
		HIV	649 (83.5)		
		General infectious diseases	128 (16.5)		
	**Length of career (years)**				
		≤5	345 (44.4)		
		6-10	184 (23.7)		
		≥11	248 (31.9)		
	**Average number of patients living with HIV treated per month**				
		≤50	461 (59.3)		
		51-100	128 (16.5)		
		≥101	188 (24.2)		

^a^The 31 provinces were located across six administrative regions.

### Self-Rated Knowledge, Attitudes, and Experience Associated With PrEP and Barriers to Prescribing

In this study, 72.5% (563/777) of the clinicians reported that they had heard of PrEP; however, only 30.8% (239/777) had an excellent or good understanding of PrEP, and 31.9% (248/777) thought that PrEP was extremely effective for reducing the risk of HIV infection. Most clinicians (635/777, 81.7%) considered that PrEP clinical guidelines were not available in China. About half (367/777, 47.2%) believed that it was necessary for clinicians to provide PrEP to HIV high-risk populations to reduce HIV infections and were, thus, defined as willing to prescribe PrEP. Clinicians were more likely to mostly recommend PrEP to serodiscordant couples (439/777, 56.5%), followed by MSM (309/777, 39.8%). Few clinicians (201/777, 25.9%) often or occasionally saw high-risk populations actively seeking PrEP prescriptions. Among the clinicians surveyed, concerns about prescribing PrEP included the following: side effects of treatment (484/777, 62.3%), promoting the occurrence of high-risk sexual behaviors (476/777, 61.3%), and poor adherence to PrEP (383/777, 49.3%) (Table 2).

**Figure 1:**
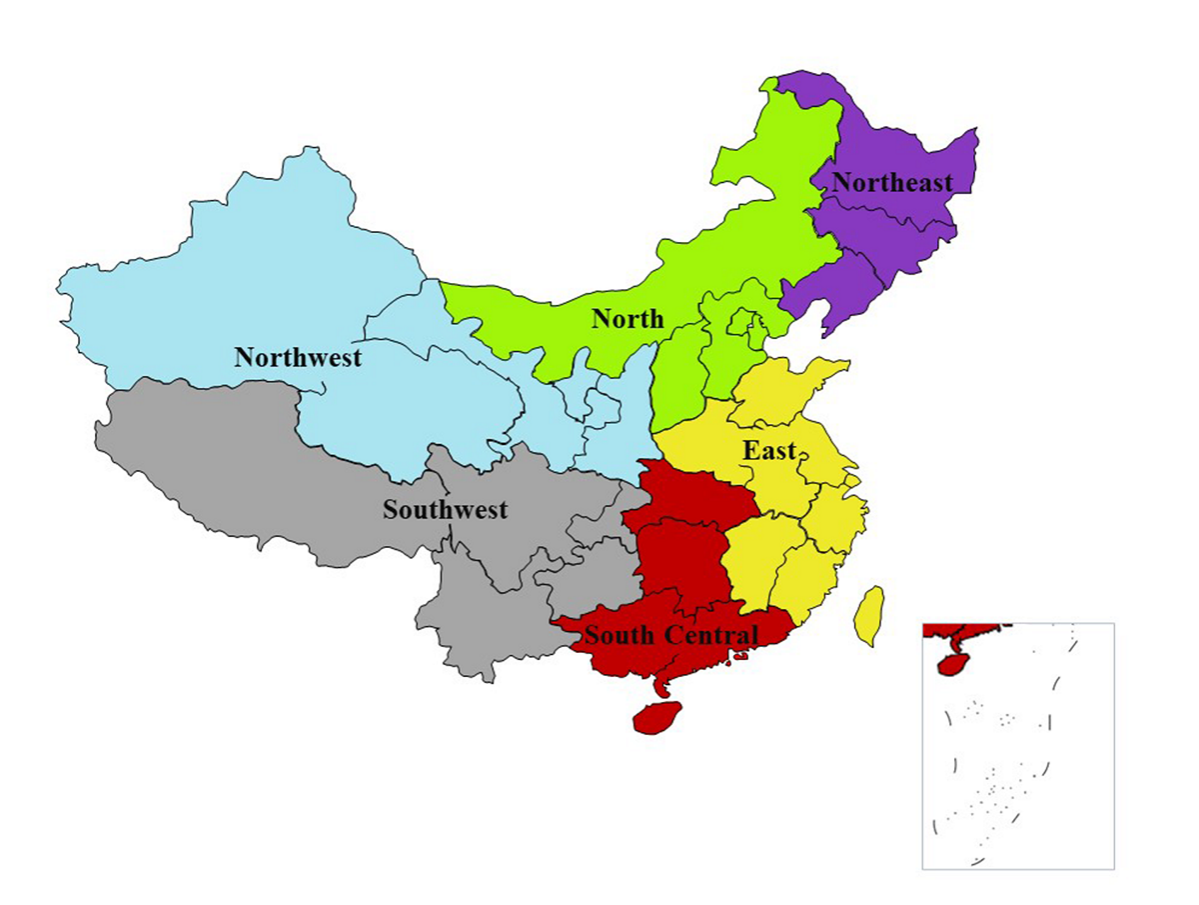
The six administrative regions of China.

Figure 1 depicts the six administrative regions of China; the percentages of clinicians from these regions who self-reported an excellent or good understanding of PrEP, who found that PrEP was necessary for high-risk groups, and who treated 50 or fewer patients living with HIV per month are shown in Table 3.

**Table 2:** Self-rated knowledge, attitudes, experience, and barriers associated with pre-exposure prophylaxis (PrEP).

Variable				Value (N=777), n (%)
**Self-rated knowledge**				
	**Heard of PrEP**			
		Yes	563 (72.5)	
		No	214 (27.5)	
	**Understanding of PrEP**			
		Excellent	86 (11.1)	
		Good	153 (19.7)	
		Low	538 (69.2)	
	**Effectiveness of PrEP on reducing the risk of HIV infection**			
		Extremely effective	248 (31.9)	
		Possibly effective	106 (13.6)	
		Not sure	419 (53.9)	
		Ineffective	4 (0.5)	
	**PrEP clinical guidelines are available in China**			
		Yes	142 (18.3)	
		No	635 (81.7)	
**Attitudes towards PrEP**				
	**Providing PrEP to high-risk groups**			
		Necessary	367 (47.2)	
		Not necessary	410 (52.8)	
	**Recommend PrEP prescription to men who have sex with men**			
		Mostly	309 (39.8)	
		Frequently	254 (32.7)	
		Seldom	55 (7.1)	
		Never	159 (20.5)	
	**Recommend PrEP prescription to heterosexual males**			
		Mostly	59 (7.6)	
		Frequently	309 (39.8)	
		Seldom	154 (19.8)	
		Never	255 (32.8)	
	**Recommend PrEP prescription to heterosexual females**			
		Mostly	58 (7.5)	
		Frequently	352 (45.3)	
		Seldom	153 (19.7)	
		Never	214 (27.5)	
	**Recommend PrEP prescription to serodiscordant couples**			
		Mostly	439 (56.5)	
		Frequently	197 (25.4)	
		Seldom	58 (7.5)	
		Never	83 (10.7)	
**Experience related to PrEP**				
	**See high-risk groups actively seeking PrEP prescriptions**			
		Often	50 (6.4)	
		Occasionally	151 (19.4)	
		Seldom	213 (27.4)	
		Never	363 (46.7)	
**Perceived barriers to prescribing PrEP**				
	**Concerned about promoting the occurrence of high-risk sexual behaviors**			
		Yes	476 (61.3)	
		No	301 (38.7)	
	**Concerned about** **increasing the risk of other sexually transmitted diseases**			
		Yes	120 (15.4)	
		No	657 (84.6)	
	**Concerned about p** **oor adherence to PrEP**			
		Yes	383 (49.3)	
		No	394 (50.7)	
	**Concerned about** **drug resistance**			
		Yes	271 (34.9)	
		No	506 (65.1)	
	**Concerned about** **side effects of PrEP**			
		Yes	484 (62.3)	
		No	293 (37.7)	
	**Concerned about the** **cost of PrEP**			
		Yes	462 (59.5)	
		No	315 (40.5)	
	**Concerned about** **reduction of resources for patients living with HIV**			
		Yes	123 (15.8)	
		No	654 (84.2)	
	**Concerned about** **lack of PrEP clinical guidelines**			
		Yes	140 (18.0)	
		No	637 (82.0)	
	**Concerned about l** **ack of drug indications**			
		Yes	108 (13.9)	
		No	669 (86.1)	

**Table 3:** Pre-exposure prophylaxis (PrEP)–related knowledge, attitudes, and medical experience of clinicians in six administrative regions of China.

Variable and regions of China		Value, n (%)
**Self-reported an excellent or good understanding of PrEP**		
	South Central (n=201)	63 (31.3)
	Southwest (n=199)	58 (29.1)
	Northeast (n=164)	49 (29.9)
	East (n=116)	39 (33.6)
	North (n=71)	21 (29.6)
	Northwest (n=26)	9 (34.6)
**PrEP was necessary for high-risk groups**		
	South Central (n=201)	95 (47.3)
	Southwest (n=199)	90 (45.2)
	Northeast (n=164)	87 (53.0)
	East (n=116)	54 (46.6)
	North (n=71)	26 (36.6)
	Northwest (n=26)	15 (57.7)
**Treated 50 or fewer patients living with HIV per month**		
	South Central (n=201)	135 (67.2)
	Southwest (n=199)	108 (54.3)
	Northeast (n=164)	90 (54.9)
	East (n=116)	69 (59.5)
	North (n=71)	40 (56.3)
	Northwest (n=26)	19 (73.1)

### Predictors of Clinicians Being Willing to Prescribe PrEP

**Figure 2:**
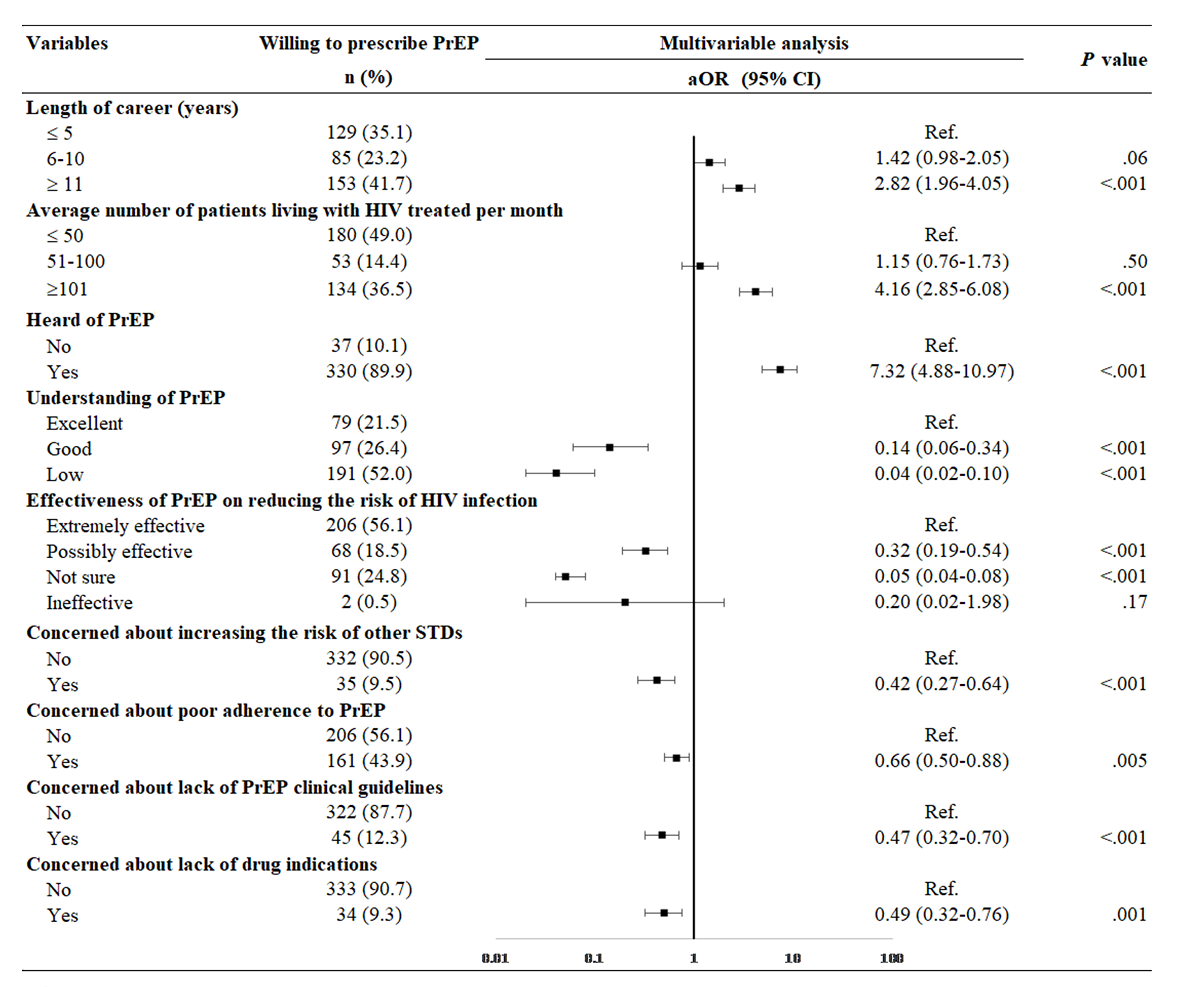
Predictors of clinicians being willing to prescribe pre-exposure prophylaxis (PrEP) (N=777). aOR: adjusted odds ratio; Ref.: reference; STD: sexually transmitted disease.

Table 4 presents the crude ORs of potential factors that could affect clinicians’ willingness to prescribe PrEP. All significant factors in the crude analysis were analyzed using multivariable logistic regression (Figure 2). After adjusting for age, gender, ethnicity, and educational background, the following clinicians were more likely to be willing to prescribe PrEP: those who had been working for more than 10 years, compared to 5 years or fewer (aOR 2.82, 95% CI 1.96-4.05); those who heard of PrEP (aOR 7.32, 95% CI 4.88-10.97); and those who often had high-risk populations actively requesting PrEP prescriptions, compared to never requesting (aOR 79.35, 95% CI 18.78-335.31).

Clinicians who had a low understanding of PrEP, compared to an excellent understanding (aOR 0.04, 95% CI 0.02-0.10), or were not sure about the effectiveness of PrEP on reducing the risk of HIV infection, compared to self-reporting PrEP as extremely effective (aOR 0.05, 95% CI 0.04-0.08), were less likely to be willing to prescribe PrEP. In addition, clinicians who were concerned about the lack of PrEP clinical guidelines (aOR 0.47, 95% CI 0.32-0.70) or the lack of drug indications (aOR 0.49, 95% CI 0.32-0.76) were less likely to be willing to prescribe PrEP.

**Table 4:** Univariable logistic regression of predictors of clinicians being willing to prescribe pre-exposure prophylaxis (PrEP) (N=777).

Variable			Willing to prescribe PrEP				Odds ratio (95% CI)		*P* value
			Yes (n=367), n (%)		No (n=410), n (%)				
**Age (years)**									
	26-39	146 (39.8)		156 (38.0)		Reference			
	≤25	0 (0)		3 (0.7)		0.15 (0.01-2.98)		.22	
	40-49	141 (38.4)		190 (46.3)		0.79 (0.58-1.09)		.15	
	50-59	76 (20.7)		55 (13.4)		1.48 (0.98-2.23)		.07	
	≥60	4 (1.1)		6 (1.5)		0.71 (0.20-2.58)		.61	
**Ethnicity**									
	Han	337 (91.8)		375 (91.5)		Reference			
	Non-Han	30 (8.2)		35 (8.5)		0.96 (0.57-1.59)		.86	
**Administrative region of China**									
	Northeast	77 (21.0)		87 (21.2)		Reference			
	South Central	106 (28.9)		95 (23.2)		1.26 (0.83-1.91)		.27	
	Southwest	89 (24.3)		110 (26.8)		0.91 (0.60-1.38)		.67	
	East	54 (14.7)		62 (15.1)		0.98 (0.61-1.59)		.95	
	North	26 (7.1)		45 (11.0)		0.65 (0.37-1.16)		.14	
	Northwest	15 (4.1)		11 (2.7)		1.54 (0.67-3.56)		.31	
**Type of hospital**									
	General	170 (46.3)		213 (52.0)		Reference			
	Infectious disease	197 (53.7)		197 (48.0)		1.25 (0.95-1.66)		.12	
**Academic title**									
	General physician	37 (10.1)		45 (11.0)		Reference			
	Attending doctor	142 (38.7)		132 (32.2)		1.31 (0.80-2.15)		.29	
	Deputy chief physician	95 (25.9)		126 (30.7)		0.92 (0.55-1.53)		.74	
	Chief physician	93 (25.3)		107 (26.1)		1.06 (0.63-1.77)		.83	
**Length of career (years)**									
	≤5	129 (35.1)		216 (52.7)		Reference			
	6-10	85 (23.2)		99 (24.1)		1.44 (1.00-2.07)		.05	
	≥11	153 (41.7)		95 (23.2)		2.70 (1.93-3.78)		<.001	
**Average number of patients living with HIV** **treated** **per month**									
	≤50	180 (49.0)		281 (68.5)		Reference			
	51-100	53 (14.4)		75 (18.3)		1.10 (0.74-1.64)		.63	
	≥101	134 (36.5)		54 (13.2)		3.87 (2.68-5.59)		<.001	
**Heard of PrEP**									
	No	37 (10.1)		177 (43.2)		Reference			
	Yes	330 (89.9)		233 (56.8)		6.78 (4.58-10.03)		<.001	
**U** **nderstanding** **of PrEP**									
	Excellent	79 (21.5)		7 (1.7)		Reference			
	Good	97 (26.4)		56 (13.7)		0.15 (0.07-0.36)		<.001	
	Low	191 (52.0)		347 (84.6)		0.05 (0.02-0.11)		<.001	
**Effectiveness of PrEP on reducing the risk of HIV infection**									
	Extremely effective	206 (56.1)		42 (10.2)		Reference			
	Possibly effective	68 (18.5)		38 (9.3)		0.37 (0.22-0.61)		<.001	
	Not sure	91 (24.8)		328 (80.0)		0.06 (0.04-0.09)		<.001	
	Ineffective	2 (0.5)		2 (0.5)		0.20 (0.03-1.49)		.12	
**See high-risk groups actively seeking PrEP prescriptions**									
	Never	88 (24.0)		275 (67.1)		Reference			
	Seldom	111 (30.2)		102 (24.9)		3.40 (2.37-4.88)		<.001	
	Occasionally	120 (32.7)		31 (7.6)		12.10 (7.62-19.20)		<.001	
	Often	48 (13.1)		2 (0.5)		75.00 (17.86-314.88)		<.001	
**Concerned about promoting the occurrence of high-risk sexual behaviors**									
	No	152 (41.4)		149 (36.3)		Reference			
	Yes	215 (58.6)		261 (63.7)		0.81 (0.61-1.08)		.15	
**Concerned about increasing the risk of other sexually transmitted diseases**									
	No	332 (90.5)		325 (79.3)		Reference			
	Yes	35 (9.5)		85 (20.7)		0.40 (0.26-0.62)		<.001	
**Concerned about poor adherence to PrEP**									
	No	206 (56.1)		188 (45.9)		Reference			
	Yes	161 (43.9)		222 (54.1)		0.66 (0.50-0.88)		.004	
**Concerned about** **side effects of PrEP**									
	No	143 (39.0)		150 (36.6)		Reference			
	Yes	224 (61.0)		260 (63.4)		0.90 (0.68-1.21)		.50	
**Concerned about** **lack of PrEP clinical guidelines**									
	No	322 (87.7)		315 (76.8)		Reference			
	Yes	45 (12.3)		95 (23.2)		0.46 (0.32-0.68)		<.001	
**Concerned about lack of drug indications**									
	No	333 (90.7)		336 (82.0)		Reference			
	Yes	34 (9.3)		74 (18.0)		0.46 (0.30-0.72)		.001	

## Discussion

### Principal Findings

This is the first cross-sectional survey to assess the knowledge, attitudes, and experience of HIV clinicians regarding PrEP in China. While the proportion of clinicians surveyed who had heard of PrEP was only slightly less compared to the proportion in other developed countries, understanding of PrEP was much lower in China. Of those surveyed, 72.5% (563/777) had heard of PrEP, which was slightly lower than the percentage in the United States (75% to 96%) [15-18] or in the United Kingdom (77%) [19]. Only 30.8% of clinicians (239/777) rated their knowledge of PrEP as good or excellent in our study, which was much lower than previously reported in the United Kingdom (80%) [19] and Canada (83.6%) [20]. We also found that 47.2% (367/777) of the clinicians were willing to provide PrEP to high-risk groups. In comparison, the pooled prevalence of clinicians who were willing to prescribe PrEP in a meta-analysis was 66% in the United States [21], and the proportion found in Canada was 45.4% [20]. As there are no PrEP guidelines in China, standardized education and guidance on PrEP is limited, leading to low awareness on PrEP. In addition, as Chinese clinicians have a shorter period of professional training and the education levels of clinicians are relatively lower in remote areas, they may be younger and less experienced when beginning their clinical work compared with experienced clinicians in western countries such as the United States [18,22]. This shorter training period and the lower education levels may limit their PrEP-related knowledge and ability to prescribe PrEP to key populations; thus, this group of clinicians in China should be given priority for training. While this survey also found that about half of Chinese clinicians were willing to prescribe PrEP, their knowledge of PrEP was low compared to other developed countries.

We found that over half of clinicians (53%) surveyed had seen high-risk groups actively seeking PrEP prescriptions, which is higher than the percentage in the United States (43%) [13]. Although Chinese MSM have a limited understanding of PrEP [26,29,30], they are willing to actively seek PrEP prescriptions, with a proportion of 75% in the relevant studies [2,25,31], while the proportion is about 60% in the United States [32].

Our study also identified the factors that promote or hinder the clinician in being willing to prescribe PrEP. Clinicians in infectious disease hospitals with longer work experience were more likely to be willing to prescribe PrEP for high-risk groups compared to those earlier in their careers. Previous studies have found that clinicians who attended more clinical training sessions were more likely to be willing to prescribe PrEP [15,16,33]. Thus, these clinicians with the longer careers could have been more likely to prescribe PrEP because they had more targeted clinical training and more extensive clinical experience over time. To increase the willingness to prescribe PrEP among those with less work experience, junior clinicians from general hospitals should specifically undergo PrEP-related training.

Concerns about the lack of PrEP clinical guidelines and drug indications were two independent barriers to clinicians’ willingness to prescribe PrEP. Clinicians surveyed who were concerned about the lack of PrEP clinical guidelines had about half the odds of being willing to prescribe PrEP compared to those who were not concerned. Thus, development and implementation of PrEP guidelines could substantially increase the number of clinicians who are willing to prescribe PrEP and support the national implementation of PrEP [15,34]. The current Chinese guidelines, published in 2018, for diagnosis and treatment of HIV and AIDS only includes the definition of PrEP [35] and does not include any information on PrEP-related inclusion criteria, laboratory testing, PrEP regimens and adherence, or potential side effects. In addition, clinicians who were concerned about lack of PrEP indications were significantly less likely to be willing to prescribe PrEP compared to those who were not concerned. At the time this study was conducted, PrEP was not approved in China for HIV prevention. Thus, clinicians could be held liable for any possible side effects of PrEP, decreasing the number willing to prescribe PrEP. This potential problem was addressed in August 2020 when the National Medical Products Administration of China approved the use of Truvada for HIV prevention. Removing this barrier should lead to more clinicians who are willing to prescribe PrEP.

The results of this survey can support the future implementation of PrEP in China. First, by determining that concerns about lack of PrEP guidelines and drug indications leads to lower likelihood of being willing to prescribe PrEP, our study suggests that China must implement and promote national PrEP guidelines. While these guidelines are currently under development, it is imperative that they are published and distributed as soon as possible [23]. Second, our finding that less experienced clinicians were less likely to prescribe PrEP, combined with previous findings that PrEP-specific training led to clinicians being more willing to prescribe PrEP, suggest that junior clinicians should attend PrEP-specific training sessions [36].

### Strengths and Limitations

This study was the first survey about PrEP-related knowledge, attitudes, and experience and barriers to prescribing PrEP among Chinese clinicians. By determining barriers in prescribing PrEP that must be addressed, our work will help promote PrEP implementation in China. We also conducted an online survey and obtained responses from all 31 provinces, while previous studies have conducted surveys only in a limited geographic area.

However, our study was limited by the small sample size compared to the total number of HIV clinicians in China, and the clinicians eligible for this study were not randomly sampled from all HIV clinicians in China. We also had few participants from certain provinces; in particular, provinces in the northwest where the proportion of clinicians who self-identified ethnically as Han was low. Therefore, our results are not representative of all HIV clinicians in China. Second, because this study was cross-sectional, we could not evaluate any causal relationship between potential factors and the clinicians’ willingness to prescribe PrEP. Third, as this survey was based on self-reported knowledge and attitudes toward PrEP, their subjective answers could be impacted by response bias. Fourth, while the ratio of HIV clinicians from infectious disease hospitals and from general hospitals is generally 2:1 in China, in our study, the ratio was about 1:1. This lower representation of HIV clinicians from infectious disease hospitals could lead to selection bias and make our results less representative of HIV clinicians working in infectious disease hospitals. Lastly, as HIV prevention regulations vary substantially between different countries, we cannot extrapolate our China-specific results to other countries.

### Conclusions

This survey found that a high proportion of Chinese clinicians had heard of PrEP, but many had limited understanding and incorrect knowledge of PrEP. The current lack of national clinical guidelines and drug indications for PrEP may lead to clinicians being less willing to prescribe PrEP. To address these barriers, the government must publish and promote national PrEP guidelines and clinicians with limited work experience should undergo PrEP-specific training to increase and promote use of PrEP among high-risk groups and reduce the risk of HIV infections.

## Appendix

Multimedia Appendix 1 Participant recruitment for the survey.

Multimedia Appendix 2 CHERRIES (Checklist for Reporting Results of Internet E-Surveys) checklist.

Multimedia Appendix 3 English version of the study questionnaire.

Multimedia Appendix 4 The distribution of HIV clinicians at the province level and economic level.

## Abbreviations

aOR: adjusted odds ratio
CHERRIES: Checklist for Reporting Results of Internet E-Surveys
MSM: men who have sex with men
OR: odds ratio
PrEP: pre-exposure prophylaxis
TDF/FTC: tenofovir disoproxil fumarate/emtricitabine
WHO: World Health Organization
