# Supplementary figures and images for: Factors Influencing Clinicians’ Willingness to Prescribe Pre-exposure Prophylaxis for Persons at High Risk of HIV in China: Cross-sectional Online Survey Study
Source: JMIR Public Health Surveill. 2021 Jun 4;7(6):e24235. doi: 10.2196/24235 (PMC8214180; doi:10.2196/24235)

**Multimedia Appendix 1:** **Survey recruitment**

**
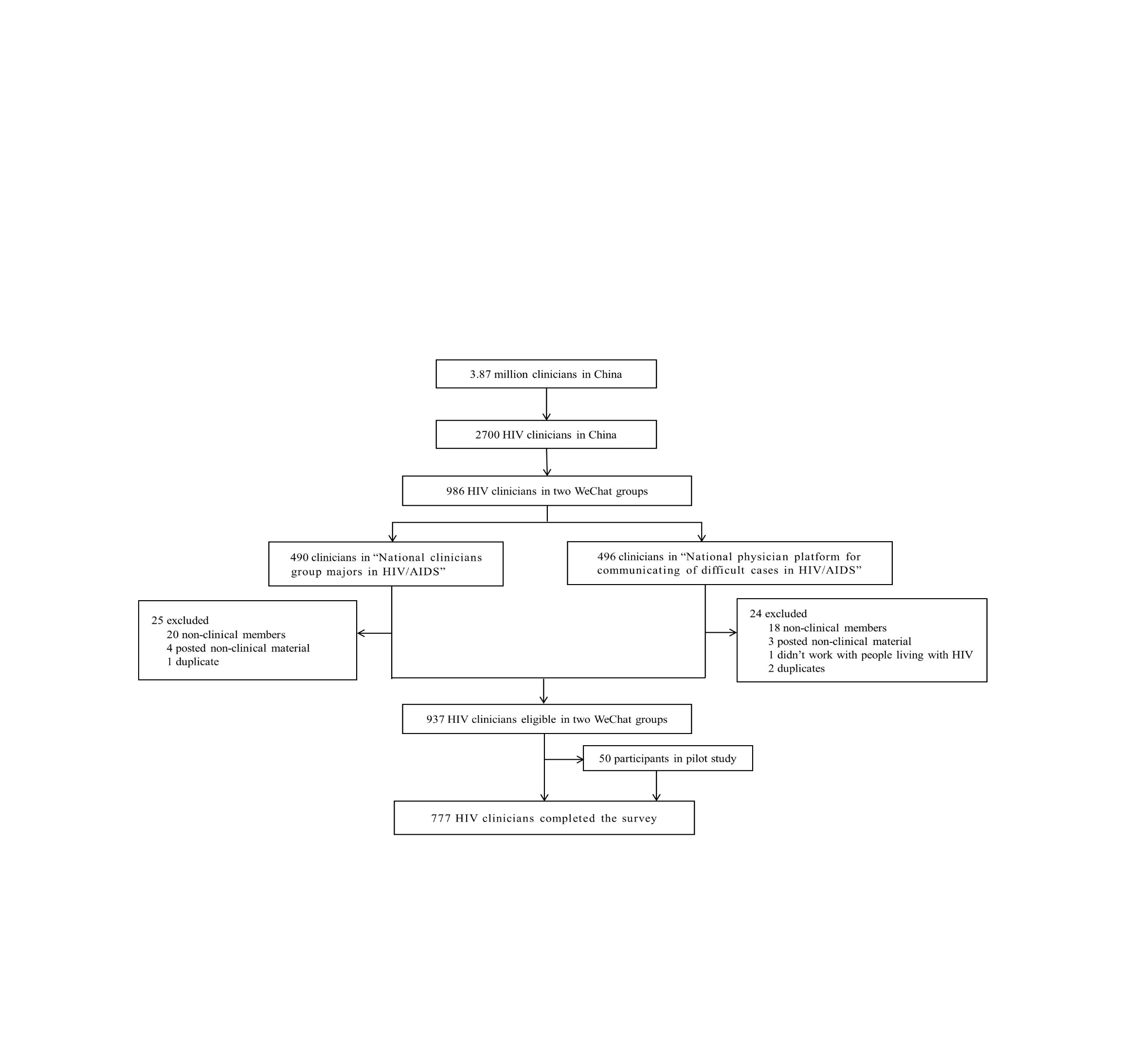
**

Supplement: Multimedia Appendix 1 [file publichealth_v7i6e24235_app1.docx]
