# Supplementary material for: Factors Influencing Clinicians’ Willingness to Prescribe Pre-exposure Prophylaxis for Persons at High Risk of HIV in China: Cross-sectional Online Survey Study
Source: JMIR Public Health Surveill. 2021 Jun 4;7(6):e24235. doi: 10.2196/24235 (PMC8214180; doi:10.2196/24235)
Supplement: Multimedia Appendix 3 [file publichealth_v7i6e24235_app3.docx]

**Multimedia Appendix 3:** **English version of the questionnaire**

**An online survey on HIV pre-exposure prophylaxis (PrEP) among clinicians in China**

**Part 1. Demographic Characteristics**

1.1 What is your department in the hospital? [single choice] *

| ○ Infectious Disease |
| --- |
| ○ HIV/AIDS |
| ○ Other  (If other, please skip to the end and submit your questionnaire) |

1.2 Do you provide diagnosis and treatment services for people living with HIV?
[single choice] *

| ○Yes |
| --- |
| ○No (If no, please skip to the end and submit your questionnaire) |

1.3 When were you born: [fill in the blank] *

_________________________________

1.4 What is your ethnicity? [single choice] *

| ○ Han |
| --- |
| ○ Mongolian |
| ○ Man |
| ○ Hui |
| ○ Uyghur |
| ○ Other |

1.5 What is your gender? [single choice] *

| ○ Male |  |  |  |  |  |  |
| --- | --- | --- | --- | --- | --- | --- |

○ Female

1.6 What is your education level? [single choice] *

| ○ High school or technical secondary school |
| --- |
| ○ Junior college |
| ○ Undergraduate or above |

1.7 What is the province of your hospital? [fill in the blank] *

_________________________________

1.8 What is the administrative level of your hospital? [single choice] *

| ○ Provincial |
| --- |
| ○ City |
| ○ County/district |
| ○ Township |

1.9 What type is your hospital? [single choice] *

| ○ Specializing in infectious diseases |
| --- |
| ○ General |

1.10 What is your title? [single choice] *

| ○ General physician |
| --- |
| ○ Attending physician |
| ○ Deputy chief physician |
| ○ Chief physician |
| ○ Other |

1.11 How many years have you provided HIV/AIDS medical services? (Less than one year will be counted as one year) [fill in the blank] *

_________________________________

1.12 How many HIV/AIDS patients did you treat in the past month? [fill in the blank] *

_________________________________

1.13 What is the estimated number of new HIV infections in China every year?
[single choice] *

| ○ 40,000 |
| --- |
| ○ 60,000 |
| ○ 80,000 |
| ○ 100,000 |
| ○ Do not know |

**Part 2. Pre-exposure prophylaxis (PrEP)**

**Self-rated knowledge, attitudes, and PrEP-related experience**

2.1 Have you ever heard of PrEP? [single choice] *

| ○ Yes |
| --- |
| ○ No |

2.2 What is your understanding of PrEP? [single choice] *

| ○ Excellent |
| --- |
| ○ Good |
| ○ Low |
| ○ Do not know |

2.3 What is the effectiveness of PrEP on reducing the risk of HIV infection? [single choice] *

| ○ PrEP is an extremely effective HIV infection prevention method | |
| --- | --- |
| ○ PrEP is a possibly effective HIV infection prevention method | |
| ○ I am not sure about the HIV prevention effect of PrEP | |
| ○ PrEP is an ineffective HIV infection prevention method | |
|  | |
| 2.4. Are there PrEP clinical guidelines in China? [single choice] * | |
| ○ Yes | |
| ○ No | |
| ○ Not sure | |

2.5 How often have you seen high-risk groups actively seeking PrEP prescriptions in the past 6 months? [single choice] *

| ○ Often (more than 1 time per week) |
| --- |
| ○ Occasionally (1 to 4 times per month) |
| ○ Seldomly (less than 1 time per month) |
| ○ Never |

2.6 How often do you provide PrEP prescriptions to high-risk groups in the past 6 months? [single choice] *

| ○ Often (more than 1 time per week) |
| --- |
| ○ Occasionally (1 to 4 times per month) |
| ○ Seldomly (less than 1 time per month) |
| ○ Never |

2.7 Do you think it is necessary for clinicians to prescribe PrEP for HIV high-risk populations to reduce HIV infections? [single choice] *

| ○ Yes |
| --- |
| ○ No |
| ○ Do not know |

2.8 Do you think it is necessary to set up a special PrEP clinic at your hospital? [single choice] *

| ○ Yes |
| --- |
| ○ No |
| ○ No opinion |

2.9 Do you think it is necessary for our country to develop an expert consensus for PrEP? [single choice] *

| ○ Yes | |
| --- | --- |
| ○ No | |
| ○ No opinion | |
|  | |
| 2.10 Do you recommend pre-exposure prophylaxis to men who have sex with men (MSM)? [single choice] * | |
| ○ Mostly recommend | |
| ○ Frequently recommend | |
| ○ Seldomly recommend | |
| ○ Never recommend | |
|  | |
| 2.11 Do you recommend pre-exposure prophylaxis to heterosexual males? [single choice] * | |
| ○ Mostly recommend | |
| ○ Frequently recommend | |
| ○ Seldomly recommend | |
| ○ Never recommend | |
|  | |
| 2.12 Do you recommend pre-exposure prophylaxis to heterosexual females? [single choice] * | |
| ○ Mostly recommend | |
| ○ Frequently recommend | |
| ○ Seldomly recommend | |
| ○ Never recommend | |
|  | |
| 2.13 Do you recommend pre-exposure prophylaxis to serodiscordant couples? [single choice] * | |
| ○ Mostly recommend | |
| ○ Frequently recommend | |
| ○ Seldomly recommend | |
| ○ Never recommend | |
|  | |
| 2.14 Do you recommend pre-exposure prophylaxis to drug users? [single choice] * | |
| ○ Mostly recommend | |
| ○ Frequently recommend | |
| ○ Seldomly recommend | |
| ○ Never recommend | |
| **Problems and barriers to PrEP** | |
| 2.15 Have you experienced any difficulty in prescribing pre-exposure prophylaxis? [single choice] *  ○Yes | |
| ○No | |
| 2.16 Is there a pre-exposure prophylaxis clinical guideline in our country? [single choice] * | |
| ○Yes | |
| ○No | |
| ○I don’t know | |
|  | |

2.17 What do you think are potential barriers to you prescribing PrEP? [multiple choice] *

| A. It may promote the occurrence of high-risk sexual behaviors |
| --- |
| B. It may increase the infection risk of other sexually transmitted diseases |
| C. Patient's adherence may be poor |
| D. It may promote drug resistance |
| E. It could lead to side effects |
| F. The cost of PrEP is too high |
| G There are no PrEP clinical guidelines in China |
| H. There is lack of information on drug indications |
| I. Due to limited resources, PrEP will reduce resources for patients living with HIV. |
| J. No barriers |
| I. Others _________________ |

The questionnaire survey is over. Thank you for your participation.
