# Supplementary material for: Factors Influencing Clinicians’ Willingness to Prescribe Pre-exposure Prophylaxis for Persons at High Risk of HIV in China: Cross-sectional Online Survey Study
Source: JMIR Public Health Surveill. 2021 Jun 4;7(6):e24235. doi: 10.2196/24235 (PMC8214180; doi:10.2196/24235)
Supplement: Multimedia Appendix 4 [file publichealth_v7i6e24235_app4.docx]

**Multimedia Appendix 4：****The distribution of HIV clinicians on the province levels and economic levels**

**Table S1. The distribution of HIV clinicians on 31 provinces (N=777)**

| **Province** | | **Economic Level^a^** | **Number (%)** |
| --- | --- | --- | --- |
| Guangdong | Level 1 | | 51 (6.6) |
| Shandong | Level 2 | | 22 (2.8) |
| Jiangsu | Level 2 | | 18 (2.3) |
| Henan | Level 3 | | 54 (6.9) |
| Sichuan | Level 3 | | 44 (5.7) |
| Zhejiang | Level 3 | | 27 (3.5) |
| Hubei | Level 3 | | 19 (2.4) |
| Fujian | Level 3 | | 11 (1.4) |
| Liaoning | Level 4 | | 80 (10.3) |
| Yunnan | Level 4 | | 76 (9.8) |
| Guangxi | Level 4 | | 60 (7.7) |
| Heilongjiang | Level 4 | | 50 (6.4) |
| Guizhou | Level 4 | | 40 (5.1) |
| Gilin | Level 4 | | 34 (4.4) |
| Chongqing | Level 4 | | 32 (4.1) |
| Hebei | Level 4 | | 19 (2.4) |
| Beijing | Level 4 | | 19 (2.4) |
| Shanxi | Level 4 | | 17 (2.2) |
| Anhui | Level 4 | | 17 (2.2) |
| Hunan | Level 4 | | 15 (1.9) |
| Jiangxi | Level 4 | | 14 (1.8) |
| Neimenggu | Level 4 | | 9 (1.2) |
| Shanghai | Level 4 | | 7 (0.9) |
| Tianjin | Level 4 | | 7 (0.9) |
| Shaanxi | Level 4 | | 6 (0.8) |
| Xinjiang | Level 4 | | 6 (0.8) |
| Tibet | Level 5 | | 7 (0.9) |
| Gansu | Level 5 | | 6 (0.8) |
| Ningxia | Level 5 | | 5 (0.6) |
| Qinghai | Level 5 | | 3 (0.4) |
| Hainan | Level 5 | | 2 (0.3) |

^a^Level 1: GDP >100000 (100 million yuan)

Level 2: 70000 < GDP ≤ 100000 (100 million yuan)

Level 3: 40000 < GDP ≤ 70000 (100 million yuan)

Level 4: 10000 < GDP ≤ 40000 (100 million yuan)

Level 5: GDP ≤ 10000 (100 million yuan)


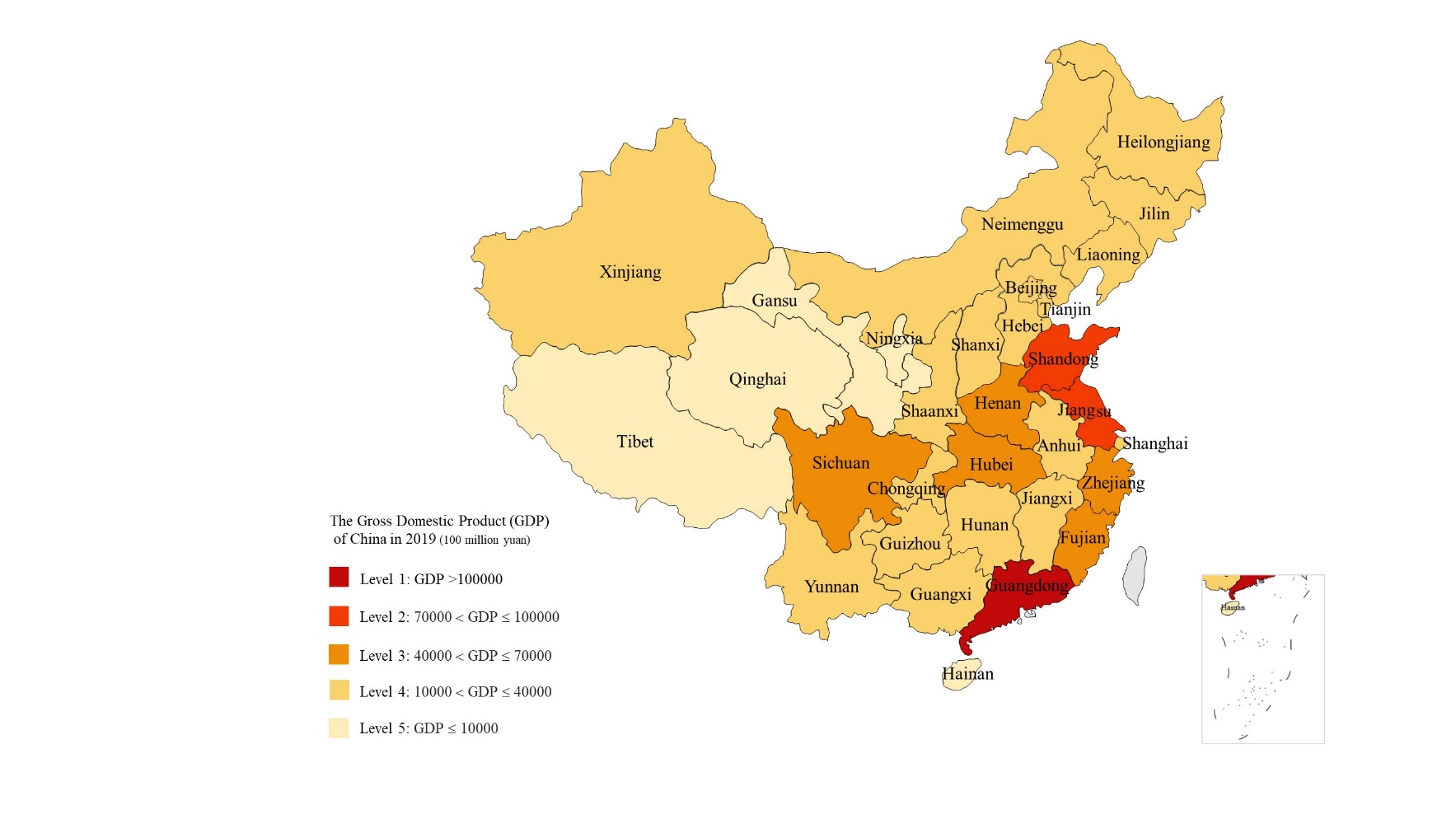


**Figure S1. Economic levels of the 31 provinces in China in 2019.**

**Figure S2. The distribution of HIV clinicians on economic levels of different provinces.**

Level 1: GDP >100000 (100 million yuan)

Level 2: 70000 < GDP ≤ 100000 (100 million yuan)

Level 3: 40000 < GDP ≤ 70000 (100 million yuan)

Level 4: 10000 < GDP ≤ 40000 (100 million yuan)

Level 5: GDP ≤ 10000 (100 million yuan)

**Table S2.** **Multivariable logistic regression for the association of economic levels and HIV clinicians being willing to prescribe PrEP (N=777)**

| **Variables** | **Willing to prescribe PrEP^a^** | | **aOR^b^ (95% CI^c^)** | ***P* value** |
| --- | --- | --- | --- | --- |
|  | **Yes (n=367)**  **n (%)** | **No (n=410)**  **n (%)** |  |  |
| **Economic levels (100 million yuan)** | | | | |
| GDP > 100000 | 27 (7.4) | 24 (5.9) | Ref. |  |
| 70000 < GDP ≤ 100000 | 20 (5.4) | 20 (4.9) | 0.84 (0.37, 1.94) | .68 |
| 40000 < GDP ≤ 70000 | 80 (21.8) | 75 (18.3) | 0.82 (0.43, 1.56) | .54 |
| 10000 < GDP ≤ 40000 | 233 (63.5) | 275 (67.1) | 0.73 (0.41, 1.30) | .29 |
| GDP ≤ 10000 | 7 (1.9) | 16 (3.9) | 0.35 (0.12, 1.02) | .06 |

Adjusted covariates: age, gender, ethnicity, and educational background.

^a^PrEP: pre-exposure prophylaxis

^b^aOR: adjusted odds ratio.

^c^CI: confidence interval.
